# “There isn't anybody else like me around here”: the insider-outsider status of LGBT residents in housing with care schemes for older people

**DOI:** 10.3389/fsoc.2023.1128120

**Published:** 2023-05-18

**Authors:** Paul Willis, Brian Beach, Jillian Powell, Alex Vickery, Alisa Cameron, Randall Smith

**Affiliations:** ^1^School for Policy Studies, University of Bristol, Bristol, United Kingdom; ^2^Institute of Epidemiology and Health, Faculty of Population Health Sciences, University College London, London, United Kingdom

**Keywords:** housing with care, aging, sexuality, gender, LGBT, older people, social inclusion and exclusion

## Abstract

The intersections between aging, social minority status and housing needs in later life is a neglected area of sociological exploration, even more so for older people who identify as lesbian, gay, bisexual and trans (LGBT). Recent sociological findings indicate that older LGBT people in housing schemes stress the importance of bonding social capital and look to other people in their social networks who reflect their identities and experiences as sources of support. In this paper, we examine the insider-outsider status occupied by older LGBT residents living in housing schemes that provide some form of care and support, for example extra care and independent living schemes. We present qualitative findings generated from a mixed-methods study of social inclusion practices in housing with care in England and Wales (UK) (2019-22). In this study 15 LGBT residents participated in semi-structured interviews (55–79 years of age) across a total of 31 interviews. Through a queer gerontological lens we examine how older LGBT people are socially situated within mainstream housing schemes in which they experience partial visibility while also encountering exclusionary pressures that locate them as “the other.” This insider-outsider status undermines the premise of housing with care schemes to provide safe, secure spaces to grow old. We discuss three core themes: (1) how LGBT residents navigate their outsider status in scheme life and how the intersection of disability and minority status amplifies this social location; (2) the exclusionary practices exercised by other residents that reinforce boundaries of sexual and gender normalcy; and, (3) the heightened importance of maintaining external social connections among LGBT residents. We conclude by introducing an alternative notion of marginal aging and expanding on the implications for housing providers, reflecting on their responsibilities for promoting and maintaining queer-friendly environments.

## 1. Introduction

The intersections between aging, social minority status and housing needs in later life is a neglected area of both gerontological and sociological exploration, even more so for studies focused on older lesbian, gay, bisexual and trans (LGBT) people. Recent findings indicate that older LGBT people in housing schemes stress the importance of bonding social capital and look to other people in their social networks who reflect their identities and experiences as sources of support (Lottmann and King, 2022). More broadly cultural gerontologists have highlighted how pervasive ideas of successful aging are entangled in depictions of heterosexual imagery that reinforce ideas of sexual normalcy in the third age (Marshall, 2018). In this paper, we build on critical scholarship on queering the notion of successful aging. To achieve this, we illustrate and examine the insider-outsider status occupied by older LGBT residents living in housing schemes that provide some form of care and support, for example extra care and independent living schemes. Housing with care schemes in the United Kingdom (UK) typically provide self-contained accommodation in purpose-built schemes that are designed to be age- and disability-friendly and that provide on-site resources including restaurants, communal areas, and access to personalized care and support (Garwood, 2016). Homecare is often provided by private care organizations contracted outside the scheme.

While health disparities experienced by older LGBT people are increasingly spotlighted in research (see Fredriksen-Goldsen et al., 2013; Fredriksen Goldsen, 2018; Westwood et al., 2020), less is known about the disparities this population experience in relation to housing security and access to affordable housing. US survey data indicates a greater prevalence of LGB adults renting and living alone compared to heterosexual adults and a lower prevalence of home ownership (Cunningham et al., 2018). This pattern of lower levels of home ownership is echoed in studies of trans citizens and comparisons between their housing situations and the general population (James et al., 2016). Similar trends are noted in the UK–lesbian, gay and bisexual (LGB) older people in England are less likely to be homeowners and more likely to expect to move in the future when compared to non-LGB older adults (Kneale, 2016).

Policy drivers on aging in place are founded on social and cultural ideals (older people's preferences to experience older age in their home and local community) and economic imperatives (financial implications of relocating to longer-term care settings) (Lewis and Buffel, 2020). A key assumption underpinning this policy position is that residents' attachment to place increases over time. However, the neighborhoods in which older people have resided long-term can be experienced as hostile or exclusionary and may not provide supportive networks to support older people as they age (Hillcoat-Nalletamby and Ogg, 2014; Lewis and Buffel, 2020). For some older lesbian and gay people “aging-in-place” may not be desirable because of their location within environments of exclusion and heterosexism (Willis et al., 2018). In the UK, older LGBT people report a greater number of community ties (i.e., participation in LGBT groups) but are less likely to report positive connections to local neighborhood (Green, 2016).

While housing with care models represent an important middle ground—providing supportive and adapted living arrangements for older people to grow old and maintain local connections central to their identity and biography—there has been little exploration of how such schemes align with the preferences and ideals of older people from gender and sexuality minoritised groups. Findings from an England-based survey suggest lesbians and gay men view residing in mainstream care provision as an undesirable future with a leaning more toward specialist LGBT-specific housing provision (Lottmann and King, 2022). However, this preference does not match the realities of current provision in the UK. At the time of writing this paper there is only one LGBT-specific housing scheme in operation for older people with plans to establish others in primarily major cities (*see* Tonic Housing, 2021). Thus, this form of specialist housing is currently not an option for the majority of LGBT people (King et al., 2021), and more accurately represents a reimagined future.

This is a crucial point to focus on social inclusion agendas in mainstream housing schemes as housing with care models are proliferating, with increases in provision and greater interest from stakeholder and policymakers to support this development. We therefore investigate this through findings generated from a mixed-methods study of social inclusion practices in housing with care schemes across England and Wales (2019–22). Through a critical gerontological lens, we examine how older LGBT people are socially situated within mainstream housing schemes in which they experience partial visibility while also encountering exclusionary practices that locate them as “the other.” This insider-outsider status troubles the dichotomous logic embedded in notions of “coming out” in later life and the dominant assumptions of housing schemes providing safe, secure spaces to grow older; both discourses require a critical interrogation of the ambivalent social position occupied by LGBT residents. This aligns with a critical gerontological focus in addressing the structural barriers and sources of inequality for diverse groups of older people and reimagining fairer, more inclusive societal arrangements for older people from minoritised groups (Torres and Donnelly, 2022).

## 2. Background literature

### 2.1. What's wrong with mainstream provision for older LGBT people?

Milligan (2009) identifies three core dimensions to the individual significance of home for older people: home as haven or protected space, home as site of identity, and home as a space of familiarity through setting and routines. All of these dimensions are impacted by physical changes to the home (such as adaptations), changes in routine and loss of control over who has access to one's home. Closer to the residential experiences of older lesbians and gay men, Kentlyn (2008) argues home operates as “a place of belonging, intimacy, security, relationship and selfhood” and a ‘safe space for the enactment of “outlaw” sexuality and non-normative gender identity' (335). Within the life narratives of older lesbians and gay men the home features as a private, protected space for performing non-normative identities and relationships free from scrutiny and the surveillance of others (Kentlyn, 2008). However, homemaking in shared living schemes with other older people can intensify forms of surveillance for LGBT residents.

The views of older lesbian, gay and bisexual people raise a number of shared concerns about mainstream care and housing provision in the UK: the invisibility of LGB identities and organizational silence surrounding non-heterosexual lives; the risk of being visible and targeted for abuse or mistreatment; marginalizing environments that restrict spaces available for LGB residents to express and display same-sex affection and intimacy; and, the anxiety of occupying residential spaces with heterosexual residents, especially for older lesbians who have lived a significant period of their lives in primarily women-majority spaces and networks (Westwood, 2016; Willis et al., 2019). A recent US survey study highlighted higher levels of worry and anxiety about future housing provision amongst older lesbians and gender-expansive people when compared to older gay men (Savage and Barringer, 2021). Differences in gender, power and social status overlapping with heteronormative arrangements compound concerns for both sub-groups. Within this body of literature is a paucity of attention to the views and wishes of older trans people and their concerns about cisnormative arrangements in mainstream housing and care environments. Results from a US survey on trans lives indicates housing discrimination as a commonly reported experience (for a quarter of 27,715 respondents) (James et al., 2016). Earlier life challenges in locating stable housing or experiencing discriminatory responses from housing providers and professionals can exacerbate older LGBT people's current anxieties about residing in age-specific housing (Redden et al., 2021). Redden et al. (2021) further suggest that housing providers need to recognize how housing histories impact on LGBT health.

Growing literature on older LGBT adults' preferences for housing in later life suggests a mixed range of views with a definite leaning toward gender and sexuality-specific housing communities. From their group discussions with LGBQ Swedish citizens, Rosenberg et al. (2018) indicate that older adults view LGB-specific spaces as an island of sanctuary and draw a symbolic boundary between general society and LGB-specific communities and spaces. However, preferences for same-gender housing identified by men and women complicate this idealized arrangement (Rosenberg et al., 2018), as echoed in other studies of LGB adults' preferences for specialized housing in later life (Westwood, 2016; Willis et al., 2019). Westwood (2017) points to a “collective homogenizing discourse” that lumps LGBT older adults' housing preferences into one model or approach and that exacerbates gender-based marginalization for lesbians and bisexual women. Explorations of specialist housing are frequently framed within a binary choice between mainstream and LGBT-specific housing that fails to take into account gender-based differences and preferences, amongst other intersecting social identities. For example, lesbian and bisexual women prefer housing options for older women over sharing residential spaces with men (Westwood, 2016). At the center of these concerns about LGBT equality and adequate housing is the interlocking of heteronormative and cisnormative discourses with mainstream policy approaches to aging and later life provision. A queer theoretical lens is critical to disrupting and unlocking this policy bind.

### 2.2. Queering notions of successful aging

Rowe and Kahn (1997) definition of successful aging incorporated three key dimensions: absence of disease (or low probabilities of), high physical and cognitive functioning and active engagement with life. Emphasis was given to the importance of active engagement in social networks and in productive roles and activities. Underpinning successful aging are notions of independence, self-reliance and active opposition to more ageist representations of older age as a life-period of decline, dysfunction and disengagement (Katz and Calasanti, 2015). Key criticisms include the difficulties of pinpointing what counts as success (complicated by little agreement on identified outcomes and measures), the need to incorporate disabilities as part of the later life experiences for many older people, and wider concerns for the emphasis on individual responsibility to age successfully. Emphasis given to individual agency and choices are at the expense of recognition of material forces such as the impact of economic resource that compound aging well (Katz and Calasanti, 2015).

The heteronormative imagery and assumptions embedded in discourse on successful aging has also been highlighted in gerontological critiques (Fabbre, 2015; Sandberg, 2015; Marshall, 2018). Marshall (2018) argues that the naturalization of heterosexuality, and the parallel consumer markets surrounding this, have become inexorably tied to ideas about positive, successful aging in the third age. Similarly, Sandberg (2015) argues that positive aging operates as a “heteronormative mode of belonging”—positive aging is wedded to heterosexual ideals and intimacies, for example, commercial depictions of erectile dysfunction within pharmaceutical discourse as a shared problem for heterosexual couples. Heteronormative ideals that situate heterosexuality as a naturalized state of being are reinforced within discussions about aging well in the third age (while discussions about sex and sexuality too-often disappear in depictions of more vulnerable living in the fourth age). Furthermore, non-confirming gender identities are rendered invisible within discussions of successful aging, as heteronormative ideals rely on dichotomous understandings of sexuality (hetero-/homosexual) and gender (male/female) (Fabbre, 2015). A focus on LGBT aging alone without a wider critique of heteronormative logic risks reinforcing and naturalizing the hetero-homosexual dichotomy rather than destabilizing it (Ramirez-Valles, 2016).

What literature that does focus on successful aging for sexual minoritised people indicates is the importance of “families of choice” alongside families of origin, having access to LGB-friendly services and the need to develop “crisis competence” through coping with adverse life experiences (Caceres and Frank, 2016). This raises the question of how LGB adults cope who are not “crisis competent,” and do not have the economic and social capital to be so, or who are rendered socially vulnerable due to having high levels of care and support needs or intensified support needs at end-of-life. Within the social networks of LGB older adults, friendships can be attributed higher social value than biological kin for older LGB adults, sometimes coined as “chosen families” (Heaphy et al., 2004) or communities of interest (Formby, 2012). In terms of household configuration, older LGB people are more likely to live alone than heterosexual peers, more likely to be childless, and are less likely to look to biological kin for support (Guasp, 2011; Lyons et al., 2013; Green, 2016). International literature suggests LGB people are more likely to look to non-related caregivers such as friends for instrumental and emotional support (Brennan-ing et al., 2014; Croghan et al., 2014). Literature from the United States puts a spotlight on the instrumental role of friends as caregivers for older LGBT people (Anderson and Flatt, 2018). Shiu et al. (2016) frame friends as “invisible carers” for LGBT people with care needs. Their survey-based research shows fewer caregiving demands but lower levels of social support for friendship caregivers in comparison to partners providing care. This study highlights the significance of social networks in bolstering caregivers' mental wellbeing.

There are two critical points that trouble the notions of “families of choice” as a reliable source of support in later life. First, the proximity in relation to friends: Kneale (2016) UK research highlights that older LGB people are less likely to see friends over consecutive days in comparison to non-LGB older adults, suggesting that LGB adults' networks may not be as equally accessible or as readily available. Second, friends are not a preferred source of more intimate and intensive care and support when needed. Lottmann and King (2022) argue that older lesbian and gay adults look more to partners and spouses for this type of intensive support and that friends represent a “fragile” source of help.

In parallel with increased critical attention on successful aging and who lies outside its narrowly defined parameters are discussions about queer aging and the application of a queer theoretical lens to disrupt dominant discourses on aging, gender and sexuality in later life. Queer theorists have historically been slow to attend to aging bodies and identities as a site of queer critique (Sandberg, 2015); however, queer perspectives make a valuable contribution toward the subversion of ageist and normative assumptions about older age and sex. Ramirez-Valles (2016) proposes queer gerontology as a new lens of critical analysis for identifying “the heterosexual norms shaping scholarship and practice in gerontology” (p. 13). As a strategy, “queer” represents a critical lens for “unmasking the ways in which heterosexual dominant norms define what it means to be an older person” (p. 13). This encompasses a critical focus on the ways in which heterosexuality as a normative discourse and practice is reproduced and privileged over non-normative identities and spaces. From this position, queer represents both a non-normative marker and a position of critique. This is founded on recognition that sexual subjectivities are generated through discourse, language and knowledge practices (Foucault, 1998).

Queer perspectives invite consideration of how other binaries resting on chronological understandings of aging (“young/ old”) operate in parallel with the heterosexual/ homosexual dichotomy to marginalize older people's expressions of sexual desire and intimacy (Hughes, 2006). Sexual/ post-sexual is an additional binary that relays assumptions about appropriate age, sex and youthfulness. Queer perspectives contribute to an awareness of “the erotic in old age” (Hughes, 2006, p. 57) by opening up recognition of and support for non-coital expressions of sexuality and different sexualities, abilities and disabilities in later life. A key critique of applying queer perspectives in gerontological scholarship is the tendency for these perspectives to emphasize the malleability of sexual and gender subjectivities and their potential for reconstruction. This does not align with corporeal experiences of aging and the ways in which experiences of physical and cognitive decline can limit scope to “reinvent the sexual self” (Moore and Reynolds, 2016).

A further significant contribution of queer theorists applicable to the current focus on older housing residents is the troubling of the notion of “coming out” that features heavily in Western discourse. Attached to this discourse is the metaphorical form of the closet as a space of shelter from LGBT-based oppression and alienation (Sedgwick, 1990). Through a queer lens, “coming out” is an interchangeable and ever-transient process of moving across the epistemological divide between visibility and invisibility. As such the closet represents a perpetually unstable space (Butler, 1991; Mason, 2002), what Fuss (1991) has described as the “infinitely permeable and shifting boundaries between insides and outsides” (4). This instability is always present within the binary logic of the in/out binary that accompanies homosexual lives and the “coming out” narrative.

In summary, there is expanding scholarship and empirical work on the housing needs and preferences of older LGBT people and the plurality of perspectives within LGBT groups on preferred housing futures. This is in parallel with policy and practice discussions about the importance of developing LGBT-specific/ friendly housing options as safe and affirming environments. However, little attention has been given to experiences of LGBT people currently living in mainstream housing schemes for older people and to whether these well-established schemes provide safe and affirming environments to grow old. Through the findings presented below we contribute new learning to this arena and further address this gap. As we illustrate, older LGBT residents do not rely heavily on the closet as a space of shelter and their accounts of scheme-life suggest a different insider-outsider status that is both highly visible and reliant on social connections that extend beyond the scheme. Our research question is, “*How do LGBT residents experience social and communal life in housing with care schemes for older people where they occupy a minority status*?”

## 3. Research design and methods

The findings presented are from a three-year mixed-methods study of social inclusion in housing with care for older adults in England and Wales. The study aimed to identify and examine social inclusion practices across a range of schemes classified as housing with care for older people (including extra care, sheltered housing, independent living), in collaboration with three housing providers. The study received ethical approval from the Faculty of Social Sciences and Law research ethics committee, University of Bristol (Reference 94582). The minimum age for resident participation was 55+ years in line with participating housing providers' criteria for residency. Here we focus on the life stories and housing experiences of 15 residents (55–79 years of age) with LGBT identities who participated in interviews. Participants were recruited through three pathways: (1) a self-completion survey distributed to residents' apartments across 100+ schemes that included an invite to take part in qualitative fieldwork; (2) flyers distributed to all residents' apartments across housing schemes participating in the qualitative phase; and, targeted email flyers that were distributed to resident groups and networks supported by one large provider, including an LGBT residents' group.

Table 1 provides an overview of participants' key characteristics and how they participated in the study. Most participants would have experienced young adulthood during the 1970s and 1980s, reflecting a generation “coming of age” that coincides with political activism amongst gay liberationists in the 1970s and HIV/AIDS activism in the 1980s (Rosenfeld, 1999). Five participants were between 70 and 79 years and therefore their life histories may straddle two identity cohorts as defined by Rosenfeld (1999)—identity as a discredited, stigmatized status (pre-liberation) and identity as a political and visible status that reflects a larger collective movement.

**Table 1:** Participant key demographic details.

**ID**	**1. Current age (at interview)**	**2. Gender**	**3. Gender same as assigned at birth**	**4. Country of birth**	**5. Religion**	**6. Ethnic group**	**7. Sexual identity**	**8. Other people in household**	**9. Highest level of education**	**10. Own or rent home**	**11. Number of interviews**
P7	70–79	Male	Yes	England	No religion	White-British	Gay	No	Advanced Diploma	Rent	3
P9	60–69	Male	Yes	Scotland	No religion	White-British	Gay	No	Blank	Rent	1
P11	70–79	Male	Yes	England	No religion	White-British	Gay	No	O levels/ NVQ	Rent	3
P12	70–79	Female	Yes	African nation	No religion	Euro-African	Gay	Yes-−1	Degree	Rent	3
P13	60–69	Female	Yes	Other European nation	No religion	Mixed Ethnic	Gay	No	Overseas qualification—diploma	Rent	3
P14	60–69	Female (trans)	No	England	No religion	White-British	Lesbian	No	No qualifications	Rent	3
P15	60–69	Female	Yes	Canada	Atheist	White-English	Gay	No	Higher degree	Rent	3
P18	70–79	Female	Yes	England	Quaker	White-English	Gay	No	Degree	Rent	2
P19	60–69	Male	Yes	England	Christian	White-English	Gay	No	Higher degree	Rent	2
P20	70–79	Male	Yes	Australia	No religion	White Australian	Gay	No	A levels	Rent	2
P21	60–69	Male	Yes	England	No religion	White-English	Other- pansexual	No	Degree	Rent	2
A11	50–59	Female	Yes	Wales	No religion	White/Welsh	Gay	No	No qualifications	Rent	1
B6	60–69	Female	Yes	Wales	Christian	White Welsh	Other—same-sex partner	No	No qualifications	Rent	1
D5	50–59	Female	No	South Africa	Christina	White-South African	Bisexual	No	O Level	Rent	1
H1	60–69	Male	Yes	England	No religion	White/British	Other—man who has sex with men	No	A Level	Rent	1

Ten participants took part in longitudinal interviews—a series of 2–3 interviews spread over a 14-month period that explored (a) participants' life stories, key identities and social backgrounds, and (b) their experiences of moving into schemes and forming new relationships with neighbors and staff. A longitudinal element was adopted to capture residents' housing experiences over time, particularly residents from minoritised groups inclusive of LGBT people, and to address the following study question: *How do residents from diverse social backgrounds experience transitions and social connections in housing with care schemes over time*? While obviously unanticipated this approach was particularly useful in capturing important changes in residents' experiences of scheme life during the first two lockdowns of the COVID-19 pandemic in 2020. The remaining four residents participated in single interviews exploring topics such as reasons for relocation; benefits of housing scheme; relationships with neighbors and staff, and participation in scheme activities and events.

All interviews followed a semi-structured format and interview guides were devised based on themes identified in the literature on housing and care for older people and in consultation with the project's advisory group (which included representatives from key organizations and charities related to older people and housing with care). Topics included: reasons for relocating; friendships and social connections within the scheme; relationships with and perceptions of neighbors; experiences of inclusion and exclusion in earlier and current life; organized activities and events within the scheme; and, relationships with on-site staff and managers. Interviews conducted between November 2019 and March 2020 were in person. Thereafter the team shifted to remote interviews through online software or telephone due to COVID restrictions and the requirement for schemes to stop external visitors. Informed consent was obtained in writing prior to interviews commencing. All participants were provided with a list of community-based support and crisis services within their locality that were mainly targeted at older people and included LGBT-specific organizations.

All interviews were recorded and transcribed verbatim. The data were imported into a framework matrix in NVivo 12, using the framework approach to analysis and data management (Gale et al., 2013). Four members of the team read a small sample of transcripts and then two of these members developed initial coding frameworks, one for the longitudinal data and one for the cross-sectional data. The frameworks included a priori categories as well as categories arising inductively. An additional category and sub-categories based on COVID-19 data was added to the two frameworks following the completion of interviews. Once the analytical frameworks were confirmed, two of the authors charted the data from the transcripts across the devised frameworks. This categorical data was then thematically analyzed using an iterative process of moving between initial coding across categories and defining and naming recurrent themes across the dataset. We adhered closely to the reflexive thematic analysis approach (Braun and Clarke, 2019), adopting a constructivist lens in recognizing the co-construction of interview encounters and themes being generated through the coding process. Emerging findings were sense checked through several presentations to different audiences including housing providers (November 2021) and members of our residents' reference group which included LGBT residents (November 2021; March 2022). Participants are assigned numerical codes when quoted to protect anonymity.

## 4. Findings

We discuss three core themes: (1) how LGBT residents navigate an outsider status in scheme life and how the intersection of disability and minority status amplifies this social location; (2) the overt and covert exclusionary practices exercised by other residents that reinforce boundaries of sexual and gender normalcy; (3) the heightened importance of maintaining external social connections among LGBT residents. First, by way of context, we briefly outline the reasons behind LGBT residents' relocation into their current schemes.

For over half the group, living with disabilities was a primary reason for relocating to their scheme. As disabled adults, participants reported problems with mobility and chronic health conditions that impacted on their everyday movements within and beyond the scheme. Disabled participants cited practical benefits of their scheme such as accessing support with daily living from carers (contracted through external homecare companies), ground floor flats that reduced the need to use stairs, flats and schemes that were (largely) wheelchair accessible, and the general comfort and security of living in an accessible apartment that was part of a bigger scheme.

Several participants stressed the importance of place, for instance having been brought up in the local area or seeking to reside close to friends or older parents. Some residents pointed to the benefits of their scheme as pull factors such as the availability of public transport on their doorstep or having access to a larger flat and a shared garden. One participant preferred to live amongst older people their “own age” while another resident cited their rural location as a sanctuary for seeking peace and quiet after a hectic and stressful working and political life.

### 4.1. Insider-outsider status of LGBT housing residents

The following accounts from LGBT residents show how interpersonal dynamics with other residents disrupt the experience of home as a protected safe space and as a site of identity validation. While relocating to their current scheme brought numerous benefits to their quality of daily living, LGBT residents occupied an ambivalent insider-outsider status within their schemes. There were some common dimensions shared with other residents, while daily practices and encounters with other residents continually reinforced their sense of social separation. Social isolation was experienced by LGBT residents on a number of levels. In the main, residents lived in heterosexual-majority environments. Most participants were the only (known) LGBT person in their housing scheme, with a small number identifying one or two other LGBT residents amongst their neighbors. In some instances, this was based on assumptions rather than direct disclosure (i.e., neighbors are not “out”). Furthermore, LGBT participants experienced social isolation by maintaining interpersonal boundaries between themselves and other residents, which reinforced an outsider status.

#### 4.1.1. No sense of community and connection

Participants reflected on the perceived lack of community life within schemes and their own sense of social disconnection from other residents. One woman (70–79 years, P18, identified as gay) drew a distinction between the quality of conversation she enjoyed with friends outside the scheme and the more “banal,” everyday conversations shared with neighbors in the scheme. Similar lines of familiarity were drawn by P21 (60–69 years, male, pansexual) stating that he would not explicitly describe himself as feeling lonely in his scheme, but he felt there was no sense of community:

*Well, I think I did that in all the schemes that I have lived in, in the four schemes. Lonely, again, I wouldn't use lonely amongst all these people, in our little flats, under the same roof. I don't feel at all a sense of companionship or community, that other word they use. … There is no sense of community, not real community*. (P21)

Here it is difficult to gauge P21's meaning behind the use of community, but the synonymous use of the term companionship suggests valued social bonds. P11, who had previously experienced homophobic discrimination from another resident in the scheme, felt that he could not be “complete” as a gay man because of his solo status as the only gay man in the scheme:

*I feel that I'm not complete here, because I seem to be like the only gay in the village, or that I know of. It's that sort of thing. It would just be nice if we could have more diversity in the scheme*. (P11, 70–79 years, male, gay)

Here P11 points to increased diversity as a solution: attracting residents from minoritised groups (for example, on the basis of sexuality, race and ethnicity) with the assumption that this will help dismantle interpersonal boundaries between residents. As a gay man, he perceived himself as lacking common ground with other residents:

*Because I'm gay and I have a different lifestyle. I don't blame them [other residents] for… They are who they are. They're talking about various things which they're interested in, television programmes and whatever. I can now talk about how ‘Coronation Street' has improved since you've got these two gay guys in it. [Laughter] Yes. It's very heterosexist, to use the terminology, here... Sometimes, things [social activities] are geared to just be exclusive to heterosexuals. …What you need is more diversity so you'd have other people you can associate more with in the scheme, but there is not that here*. (P11)

Another gay man (P7) reflected on how he shared some connections with other residents in his schemes (“on the same wavelength”) while some of his neighbors harbored “antigay views”:

*They're people with which you could say we're roughly on the same wavelength, I think, very roughly, and quite a few of them are very antigay. Some of them are. I say, “For God's sake, get over it, you know. There are quite a few gay people around, and it's time you accepted that.”* (P7, 70–79 years, male, gay)

H1 (60–69 years, man who has sex with other men) spoke of being isolated in his scheme, including prior to the pandemic. He explained that nobody was friendly in his scheme and not having common interests with other residents made him feel isolated. He was also concerned about other residents' attitudes toward people living with HIV:

*Yes, that whether I like it or not, I am very isolated…. People not liking people. Oh, they might say yes, but ___ problem with people having HIV and people being gay. They might say that they don't have a problem with it, but one thing is saying it, another thing is doing it*. (H1)

For gay men with HIV, there are unique challenges in constructing a positive identity as an older person due to the prevailing social stigma of HIV and the uncertainty about the future (Ramirez-Valles, 2016). “Gayby boomers” are the cohort most affected by HIV due to the high number of deaths experienced within their social networks and the scale of grief and loss (Rosenfeld et al., 2012; Ramirez-Valles, 2016). These factors compound concerns about exclusion in housing schemes where HIV is often an invisible topic or provokes fear: “*The people living here are very afraid of HIV. And gay [people].”* (H1).

Several participants conveyed their agency in actively excluding themselves from social activities within their schemes—they framed these decisions as active choices rather than examples of exclusion. Another man living with HIV actively avoided mixing with other residents in his scheme as he felt they were too different in age group and was not interested in the social activities run within the scheme: “*I don't mix with anyone in here... Plus, the residents here are sort of 80–90 years old*” (P9, 60–69 years, male, gay).

In relation to feelings of exclusion, P18 expressed her concern about the housing provider letting apartments to too many (heterosexual) couples—she frames this as a source of discrimination against LGBT residents who are more likely to be single in later life:

*But over the last four or five years, [housing provider] have this policy of letting to couples. Now, that mitigates, it discriminates, against LGBT people. Because most of us, at our age, are single. And it's something we've been bringing up for years, and they won't change it. … I'm very much on my own here. And the couples that are moving in are actually- They're mostly probably- I know the moving-in age are 55, but they're all sort of that younger age range, and they don't mix, they haven't come here to be part of a community. So if we see them, we say hello, but the couples don't join in anything*. (P18, 70–79 years, female, gay)

P18's comments suggest there is social chasm between “younger” residents in heterosexual relationships and herself as a single older lesbian. Younger couples residing in the scheme were perceived as less interested in creating a sense of community with other residents. This chasm is widened when considering intersecting differences across age, relationship status and sexuality.

#### 4.1.2. Social voids on the basis of ethnic and national identities and age-based differences

In reflecting on their “outsider” status some participants pointed to intersecting social differences on the basis of ethnicity, nationality and age that compounded the social void experienced between themselves and other (perceived to be heterosexual) residents. P12, a woman of Euro-African background living with her partner, explained that her living situation did not make her feel excluded but believed that their household was perceived as an oddity by other residents. This potentially generated envy amongst other residents who were not in couples:

*Well, yes. Obviously, we are odd. First of all, I'm the only one in a couple relationship in the whole building. Sometimes people pass remarks such as, “Yes, because you know I come home and I'm alone, and you go home, and you have somebody.” There's sometimes a bit of envious, snide remarks. People forget that when they were having babies and taking care of their husbands, I was alone. … Now I'm lucky I have company. I've heard comments. We are odd because we're a couple. We're two women. We're foreigners. When my neighbor said… I say, “I know that people think the bloody foreigners come here and take the NHS and take the flats.”* (P12, 70–79 years, female, gay)

P12 points to the relationships that many of her neighbors experienced in earlier aspects of their lives while she lived a significant part of her earlier life single and alone. Her reflections on their relocation to the scheme also highlight the intersection between ethnic, national location of birth and sexual minority status that compounds a sense of being perceived as “odd” to others. When her partner initially came to live with her, she felt that there was shock amongst other residents because of two women living together from different nations outside the UK:

*They don't say it, but when I first said to everyone, “My partner's coming, and she's a woman,” I think maybe there was a bit of shock. I think that there's this unspoken thing that, “Ah, yes, so she brought another foreigner here.”… It's also strange here, going back to feeling an outsider, a bloody foreigner..*. (P12)

P12 elaborated that, while she does not feel excluded by other residents as a “foreigner,” she does not feel included either. Another resident (P13) who identified as a gay woman of mixed ethnic background discussed how other residents in her scheme frequently talked about “foreigners” at social gatherings as they expressed complaints about non-white people and immigrants in UK territory:

*Yes. It's always people complaining about the foreigners on every get-together-… See, then I can't keep my mouth shut. I let them have it with more than one barrel. That doesn't always go down well. It [offends me], yes. It offends me on behalf of those people as well*. (P13, 60–69 years, female, gay)

Some participants attributed differences in views and values between other residents and themselves founded on differences in age. Other residents were positioned as “older” and therefore holding fixed beliefs that were described as conservative and oppressive. P19 pointed to the homophobic attitudes of other residents of a “certain age” and attributed this as based on the majority of residents being “elderly”:

*But I got rather annoyed with a couple of people who actually were very homophobic. Because of the elderly nature of the place, it was 55 and over- And I did have to mention it to a manager a couple of times, that I was very disappointed with some of the people's attitudes*. (P19, 60–69 years, male, gay)

Similarly, P14 commented on how “how nasty” older people could be within her scheme, describing some of her neighbors as “vindicative”—this did not align with her expectations of people in retirement: *I thought people were supposed to be retired and they're supposed to be mellow. Not this lot*. (P14, 60–69 years, female trans, lesbian). Despite this perception, she had established good friendships with a small number of neighbors, other women, two of whom she assisted with looking after their dogs. She had also assisted some neighbors with solving IT problems in their homes.

#### 4.1.3. Points of inclusion and connection

LGBT residents elaborated on the social interactions that made them feel included—social encounters that reinforced an insider status. Managers were one key source:

*I like having them around, because it gives a legitimacy to it being a communal scheme, as opposed to just a block of flats that people live at. … I don't get one, but if you want an early-morning call or a morning call, they give you a buzz and, “Are you okay? Have you made it through the night?” sort of thing*. (Laughter) (P15, 60–69 years, female, gay)

Neighbors were another key source of inclusion. LGBT residents recounted positive experiences of neighbors inviting them into their apartment or seeking their assistance with a practical task. As a trans woman, P14 appreciated the ordinary conversations she had enjoyed with other residents in communal areas:

*I meet people out in the laundry room. A lot of the women, because there are more women than there are men. You meet them out there and you just end up having a good chat and a talk and stuff like that. …. They don't treat me any different*. (P14, 60–70 years, female, trans, lesbian)

For P14 the laundry was also experienced as a paradoxical space as she later encountered transphobic materials distributed through the laundry. Another resident had printed leaflets targeted at her and left them in a communal area for others to access: *And one day, I went into the laundry room, it was out here, and I found this. A whole pile of them. A whole pile of [transphobic] leaflets* (P14). The manager had responded promptly and addressed it with the resident concerned. P14's account highlights the unpredictability of communal areas as spaces of connection and affirmation—this further disrupts scheme life as a “safe haven” to grow old.

Regular conversation fostered a sense of inclusion. P15 (60–69 years, female, gay) reflected on the friendships she had established within the scheme and what she valued about these friends: *Actually, I've made friends... We have discussed it and actually decided it's not about what people are. It's about how you think and what you value*. Her comments convey reflections on the significance of friendships that transcend identity-based differences and find common ground in shared values.

### 4.2. Exclusionary practices exercised by residents

Housing with care schemes are intended to provide secure, supportive environments for older people that promote independent living while counter-acting social isolation. The majority of participants had experienced exclusionary pressures in their current and previous scheme. These pressures were in the form of practices exercised by other residents that vilified and marginalized LGBT residents on the basis of their sexual and gender identity, or LGBT individuals and groups more widely. Such practices severely compromise the safety and wellbeing of residents and compound, rather than reduce, isolation by generating boundary markers between majority residents and LGBT individuals.

Not all such practices were experienced in a direct, targeted way. Some experiences were more covert and difficult to read as related to residents' sexual and gender identity. While the majority of participants were “out” as LGBT to other residents and staff, one participant had chosen to share this information to a select few residents, including a former resident who also identified as non-heterosexual. The former neighbor had unfortunately outed her to other residents. P15 reflected on her preference to be known as a “good neighbor” rather than the “weird woman who lives down the corridor.” This partly drove her decisions not to be out to many people in the scheme:

…*It is a nice scheme and, actually, I know a lot of people. I still drive, so I'm the go-to person if people need a lift or whatever. …I'd much rather, as I said earlier, be known as being a good neighbor. You know, “Oh, you can ask [Person 1] because she's okay.” Rather than thinking they have to filter through all my labels before they get to me*. [Laughter] (P15, 60–69 years, female, gay)

Her comments suggest she is valued as a fellow resident and neighbor and that her identity as a gay woman would potentially spoil her valued role within the scheme.

Several residents anticipated homophobic treatment even though they had not experienced this within their scheme. A11 preferred to be private about her sexual identity. She believed that some residents would hold discriminatory views stemming from their age and the era in which they grew up:

*I think people would have a problem with it, because in their days you used to be beat up. Because of the age. Being gay in those days was terrible. They would really lay into them, you know*. (A11, 50–59 years, female, gay)

P20 explained that there had been incidents of homophobia in his scheme. Consequently, he tended not to be too open about his sexual identity to other residents:

*I tend not to say too much. I just think given the age of people here, it's very unlikely that they weren't homophobic at some point in their lives. You'll have to wait for another 30 or 40 years, if there are such places still standing like the one I'm in, before it becomes that there's no more need to have an LGBT inquiry or group, because it's just not important and it's just not an issue anymore*. (P20, 70–79 years, male, gay)

Four participants shared experiences of targeted homophobic treatment within their scheme. Most of these experiences occurred at the beginning of their residency—this may indicate an easing of exclusionary pressures as relationships between residents become established. P11 had experienced homophobia from other residents in both his previous scheme (same organization) and current scheme. During the three waves of interviews however, he explained that scheme life had changed and other residents were more accepting of him as a gay man. When he was initially invited by staff to coffee mornings, he felt other residents' conversations were not appropriate:

…* that's where these unfortunate comments came because we were all chatting away about this, that, and the other. I thought, “This is not very appropriate for me.” Then, of course, we've got one woman who… She's a bit odd. She's not too bad, I get on with her okay now really. I think she meant well, but it's weird. Somebody came along and sat in the lounge. She said, “He's gay.” She wasn't saying it nastily. I thought, “Why mention it?”* (Laughter) (P11, 707–9 years, gay)

He believed some of the residents were homophobic and did not value him as a gay man, even though more recently they do not say anything specifically to him. Again, he situated their comments within the context of their (older) age and stemming from wider generational views:

*They've had homophobic, racist, sorts of comments which are not enlightened because they don't understand because of the way they are. You know, they're getting on a bit and they've lived a different life to me*.

In his second interview (four months later), P11 stated how certain residents had become more accepting of him and one particularly homophobic resident no longer visited the communal lounge. This highlights the merits of a longitudinal interview design for gleaning shifts in social dynamics between residents over time.

Homophobic, and in some instances transphobic, treatment in schemes was not commonplace but typically the actions and expressions of particular individual residents. As a trans woman, P14 had previously been aware of being called names by one resident, but this was never to her face:

*She [neighbor] started telling me stories about how I was called ‘ladyboy' and all this kind of stuff by some of the residents. I said to her, “I don't care, as long as they don't say it to my face because then they're in trouble because I will call the police.” And I've probably been referred by the people here by probably similar things, but anyway*. (P14)

Experiences of exclusionary treatment were less prominent in interactions with scheme staff, but it was raised by two participants. P18 (70–79 years, female, gay) spoke in detail about the difficulties she had encountered with her scheme manager over a number of years. She described being targeted and bullied by the manager which she had attempted to address by speaking to a more senior member of management—she was not hopeful of a positive outcome. She framed this targeted behavior as homophobic. She felt discriminated against by the scheme manager at one of the summer parties—other residents had been told they could bring along several guests while she was told otherwise: *Most of them said they could have a couple of friends, and I was told I could only have one friend. In the end, I didn't invite any of my friends. I just didn't want them to feel humiliated*. (P18)

These experiences had not deterred her from hosting LGBT social events within the scheme, including events marking LGBT History Month in which external visitors had attended along with scheme residents. H1 had found it difficult to access care support from one company of carers (external companies were often contracted to provide regular homecare to residents with care and support needs—a common feature of housing with care schemes). He had found carers were reluctant to enter his house and assist him in person, attributing this to the stigma of HIV:

*I find people, some of the so-called caring team here in [Company 3], being afraid sometimes of having contact with me. … And the medical director says, “You can come in.” And the person of the team of carers at [Company 3] say, “No, I don't want to go into the flat.”* (H1)

Fortunately, there were other companies he could access for daily assistance, and the above company was only requested during emergencies. However, the need during emergencies implies a more heightened state of vulnerability in which the care response of others is critical.

### 4.3. Heightened importance of social connections outside the scheme

A common thread running through LGBT residents' accounts was the importance of connections to friendships and groups external to their scheme that reflected aspects of their own identities, life experiences and interests. This reinforces other findings from studies of LGBT residents in mainstream housing schemes where bonding social capital is attributed high value (Lottmann and King, 2022). Group connections included LGBT-related groups and associations, political parties and artistic/creative networks. This was a more tangible thread in the interview accounts of LGBT residents than other residents participating in the study. Furthermore, the acute impact of COVID-19 restrictions inevitably led to some participants reflecting on the social contact they had valued prior to the restrictions. P18 (70–79 years, female, gay) felt connected to numerous friends outside of the scheme through the telephone and through messaging apps on her mobile device but missed being with those friends in person, particularly through her local art group: …* But it's having people near me. When we sit in group doing the art, we're all gay, we're all doing our bits of art, and it's just that nice atmosphere when you've got other people around you who are part of you, really*. (P18). This last comment emphasizes the importance of sharing the company of people that reflect aspects of her own identity and self. For P15, although she is one of the youngest in the scheme compared to the majority of older residents, she did not feel isolated as she maintained a number of friendships in the local area who she saw regularly (pre-COVID) and was able to get out and about in the community:

*Quite honestly, the sitting around, drinking tea, and all the rest of it, I'm quite happy to be left out of. (Laughter) As I say, I've got friends who are still working, and actually out and about in the community, and so I see them as well. Actually, I've got the best of both worlds, really. I can be out and about, and buzzing, if I feel like it*. (P15, 60–69 years, female, gay)

P7 (70–79 years, male, gay) was actively involved in local groups that had been part of his life for a long time and from which he drew both support and friendships, including an alcohol and substance misuse support group and an opera appreciation group. P18 had considered moving out of her scheme due to her concerns about the behavior of the manager and other residents, but there were numerous pull factors that kept her anchored in her current home, including LGBT friends in the local area: *And I keep thinking, “I'd love to move away, I'd love to move somewhere else,” but it took me a long time to make friends, make LGBT friends, here, and that was a long time ago. And the older you get, the more difficult it is to get out and meet people*. (P18, 70–79 years, female, gay)

Living with disabilities and related health care needs impacted on residents' capacity to access external groups and organizations important to them and in some instances shaped the modes through which participants could engage with such groups. P13 (60–69 years, female, gay) lived with emphysema and mental health difficulties (depression, anxiety), which compounded her isolation during the first pandemic lockdown and restricted her social activity beyond the confines of her apartment. The majority of her friendships were with people external to the scheme who lived some distance away but remained close friends: *One of my closest friends, she lives in [Place 2], and she is like my sister. She used to come over and we'd spend Christmas together, and she stayed in the guest room and things like that*. P13 likens her close friend to a sibling. She had also established a new friendship via an online neighborhood meeting application; they had met in person and continued their friendship during the 2020 lockdowns. Online applications and digital communication became even more important for accessing essential services (for example, grocery and prescription deliveries) and for keeping in touch with significant others, namely friends.

Inevitably the lockdown restrictions introduced in 2020 severely reduced participants mobility within and outside the scheme and had an impact on social contact with others. H1 was a wheelchair user and relied on carers to assist him with everyday mobility outside of his flat—this had hindered him from participating in social activities organized within the scheme and accessing drug and alcohol recovery support groups that were important to him outside the scheme. His isolation and lack of mobility were accentuated during the first pandemic lockdown:

*All the residents are difficult at the moment because of the COVID-19. I couldn't get out of my flat unless somebody takes me out. I wait for somebody to take me back to the flat*. (H1)

Staying connected to others relied heavily on access to transport for travel. P11 (70–79 years, male, gay) explained that he did not feel isolated but would like to travel about the local community some more, like he had enjoyed previously when he was part of a number of different community groups, including a local political party group and LGBT group. For LGBT residents the implementation of age-based restrictions during the early phases of the pandemic severely curtailed access to external groups and networks that were critical to their social wellbeing.

## 5. Discussion

Findings from this study provide a unique viewpoint on how LGBT people in later life negotiate shared living environments in which there are very few studies examining the social dynamics between LGBT residents and other residents in housing with care schemes for older people. This a key topic to expand on in gerontological scholarship as the provision of such schemes is likely to grow, at least in Western nations where such schemes are well-established.

As a consequence of these normative pressures, LGBT residents occupy an ambivalent insider-outsider status. They retain the sanctity of their own apartment while communal spaces, such as laundries and lounges, are sites of both connection and exclusion on the basis of sexuality and gender identity. The exclusionary practices experienced across scheme life reinforce heteronormative and cisnormative social arrangements and isolate LGBT residents. It is the ways in which LGBT residents are made visible in their interactions with other residents that warrants deeper attention, more so than whether they are “out” or not. Participants' descriptions of their negative interactions with other residents reflect subjectivities of sexual and gender “oddity,” LGBT identities as a source of irregularity, a social threat (amplified through intersections with a HIV status), or more directly a target of hostility and derision. These subjectivities of abnormality and social disruption highlight the level of surveillance LGBT residents experience under the heteronormative and cisnormative gaze of mainly other residents; the communal nature of scheme life undermines the safety and security supposedly afforded by private apartments on site (Kentlyn, 2008). Their stories of scheme life reflect a form of partial inclusion—an unsettling status that heavily shapes their relationship to and dynamics with other residents and staff.

Residents in this study reflect a new inside-outside binary, one wavering between inclusion and exclusion, which is unstable and that undermines the promise of housing with care to provide safe, secure and person-centered environments to grow old. They are in a perpetual state of becoming included, reflected through good relationships with some neighbors and points of connection, that is subject to disruption when other residents from a social majority position remind them of their partial status through everyday expressions and acts of exclusion. Returning to Milligan's (2009) framework, these experiences disrupt the symbolic value of home as a haven or protected space for older LGBT people. However, their agency in maintaining external connections with LGBT-specific social ties and groups and maintaining some neighborly bonds within schemes highlight their efforts in homemaking as site of identity, albeit under heteronormative and cisnormative pressures. External social connections that bring friendship, support and social validation become even more important in this context. These connections show agentic decisions in retaining significant others and social networks [what King and Cronin (2016) highlight as bonding social capital] that remain supportive and accessible, for the most part, post-relocation into scheme life.

Key findings also trouble ideas about successful aging for LGBT residents in the third age. A fundamental dimension of Rowe and Kahn's (1997) definition was active engagement, yet the exclusionary pressures from the insider-outsider position among LGBT residents impose unique barriers for full engagement and participation within their schemes. While some residents were able to find engagement outside of their schemes, this strategy was not foolproof, as the shock of COVID-19 lockdowns demonstrates. It may also be argued that residents who conform more to the heteronormative and cisnormative assumptions prevalent in schemes do not experience the same insider-outsider exclusionary pressure that LGBT residents experience, enabling them to achieve greater engagement and subsequent “successful” aging.

LGBT residents in our study sought social engagement through external networks, highlighting the importance of LGBT social groups and ties. When an outsider status is intensified through exclusionary pressures these groups and networks become even more important, but may not be readily accessible when social distancing measures are in place or when disabled residents do not receive sufficient support to access such networks. Other UK studies have highlighted how older lesbians and gay women maintained social connections online during the pandemic restrictions (Westwood et al., 2022), echoing similar findings to our study. We are reluctant to apply the label of “families of choice” to these bonds without further examination of the strength and closeness of these relationships—we do not want to promote this well-established concept without further testing it empirically. Nonetheless, these queer associations are important sources of “bonding capital” (King and Cronin, 2016) that partly counteract the exclusionary pressures experienced in housing with care schemes.

Within the findings exclusionary pressures are not experienced on the basis of sexual and gender minority status alone. The social voids between LGBT residents and other residents are co-constituted through intersecting differences that encompass perceived age differences and differences in ethnic and national background. The questioned status of “foreigners” in some schemes brings another layer of boundary-making in scheme life where racial, ethnic and sexuality-based tensions are openly expressed by residents and sometimes challenged. Hill Collins and Bilge (2016) propose six core ideas informing an intersectional lens, four of which we apply here. First, *social inequality*—the exclusionary pressures exercised based on intersecting differences render social chasms between residents and can lead to differential treatment in scheme life. Second, *power*—these forms of unequal treatment and exclusion expose the exercise of power by some residents to amplify the outsider status of marginalized residents. Third, *relationality*—bringing a focus on the interconnections between ethnic, national and sexual difference and the ways in which these intersections compound outsider status. And fourth, *social context*—the importance of recognizing how mainstream housing schemes can both support diversity in aging while also reiterating social differences and inequalities that marginalized residents may have experienced at earlier points in their life course.

It is important to note that LGBT residents are not immune from these forms of boundary-making as their accounts also highlight the expression of othering practices grounded in perceived age-differences. This is where Collin and Bilge's fifth theme of *complexity* is also useful for recognizing parallel practices of othering in operation that associate, and intersect, with ageism. Applying a queer gerontological lens (Ramirez-Valles, 2016) heightens sensitivity to less tangible binaries present in LGBT residents' accounts of differences between themselves and other residents. In some of their accounts other residents in the scheme are positioned as “too young” and therefore not a source of connection or “too old” and therefore fixed in their identities and mindsets. The latter position represents a distinct binary present in some participants' explanations—a binary in which liberal values and beliefs about non-normative sexualities and genders are perceived as attributes belonging to younger, “youthful” minds while older people are viewed as lacking capacity to change their more restricted and static beliefs about sex and sexuality. This binary logic also diminishes recognition that heterosexual- residents may have experienced non-normative sexual encounters and relationships at earlier points in their life course or in current relationships; non-normative sexual and gender subjectivities are not the sole domain of LGBT individuals.

To conclude, successful aging should not be the driving framework for understanding LGBT aging in mainstream settings, inclusive of housing with care schemes, not least because of the alignment of successful aging with normative expectations about how later life should be experienced. Two of the dimensions of Rowe and Kahn (1997) definition for successful aging are freedom from disease and high physical and cognitive functioning. People living in housing with care are more likely than others to have some form of morbidity and/or physical impairments and accordingly more likely to require home-based assistance with daily tasks. If conceptualisations of successful aging introduce exclusionary principles on these lines, it may be that the insider-outsider status experienced by LGBT residents in housing with care would further extend to social experiences of all housing with care residents when juxtaposed with older people living in general, mainstream housing. Indeed, the individual agency focus of theories like successful aging fail to incorporate the broader factors that shape interactions, engagement, and participation in society. Our findings suggest that “othering” persists in housing with care, leading to exclusionary pressure on LGBT residents over which they have no autonomy or independent control.

Instead, we propose an alternative lens of *marginal aging* that adopts a queer gerontological lens (Ramirez-Valles, 2016) for identifying ways in which heterosexual and gender-based norms are reinforced and maintained in older people's housing and care settings and that bolster exclusionary pressures. The emphasis on marginality takes into account the insider-outsider status of LGBT residents and the heteronormative and cisnormative exclusionary pressures enacted by other residents. The modern metaphor of the closet is not a defining aspect of participants' accounts of resident life as they are visible and “out” to others as LGBT within schemes. However, the ever-transient process of moving between visibility and invisibility (as discussed by Butler, 1991 and Fuss, 1991) remains a prominent dimension to their experiences as their queer visibility is challenged by other residents through exclusionary pressures and attempts to render them invisible and therefore marginal. The status of marginal aging is not a position of powerlessness—LGBT residents in the findings above point to their attempts to challenge the exclusionary views and expressions of other residents (and in one case a scheme manager) alongside the bonds they have built with some neighbors within scheme life. To return to Foucault (1998) power is not wielded or possessed by one party (or resident sub-group) over another but instead exercised by all residents in a complex field of shifting social relations and power dynamics.

## 6. Limitations

There are some limitations to the research design. First, later recruitment waves relied more on existing residents' groups, including an LGBT residents association. While this targeted the intended population it may have generated more interest from residents who are actively engaged in scheme life and bring a shared concern about enhancing social inclusion. LGBT residents who are more isolated in schemes and not as well-networked may be overlooked. Our initial recruitment strategy involved visiting schemes in person and speaking to groups of residents about the study however pandemic restrictions removed any chance of continuing this approach. Second, we gathered some information about participants' earlier lives prior to living in their current scheme but this was limited due to the study's chief focus on social inclusion in older people's current residences. This prevented drawing out comparisons between earlier and current home-life and wider relationships with neighbors, place and local community. Finally, the findings are generated from a small, non-representative sample that is weighted more toward the accounts of “younger-older” people.

## 7. Concluding comments

The accounts of older LGBT residents shared in this paper highlight the tangible benefits of living in housing with care, particularly for those aging with disabilities. Housing with care schemes are a well-established form of provision that is likely to be a more accessible option for the majority of LGBT people in the UK as they age. However, its accessibility in design does not bring with it the promise of inclusion, at least not fully, particularly for LGBT residents experiencing marginal aging. What is sorely needed is a reimagining of mainstream housing schemes that eases, if not removes, hetero- and cisnormative pressures and counters boundary-making practices between residents.

A critical challenge for housing providers is to unpick the heteronormative and cisnormative expectations of other residents and generate living environments that support more equal power relations and diminish the marginal status of LGBT residents. We would argue that housing managers and staff have a critical role to play in both recognizing LGBT residents as experiencing marginal aging and providing additional support at the individual and organizational (scheme-wide) levels to enable housing with care schemes to be affirming environments for queer lives to flourish. Without this the process of marginal aging will be amplified.

There is a need for ongoing dialogue between staff and residents about what it means to live in an inclusive neighborhood in which the differing identities and life histories of residents, including sexual biographies, are recognized and affirmed. Alongside this is a demand for zero-tolerance approaches to exclusionary acts and expressions targeted as minoritised residents. The ongoing education of staff about normative ideas attached to sex, gender and aging and how these obstruct more affirmative approaches to aging and equality is one priority area. This needs to go beyond “working with minority groups” approaches to training, and instead, give more attention to privileged discourses of heteronormativity and cisnormativity and how these intersect with other sources of social division and shape power dynamics within the micro-neighborhoods of scheme life.

One way forward is the continued involvement of external groups and organizations in the social life of schemes—groups and organizations that represent minoritised communities and reflect different generational experiences while bringing a shared commitment to supporting social bonds between residents. As specialist forms of housing are gradually starting to emerge, future research comparing the housing experiences of LGBT people in LGBT- specific provision with the experiences of those in mainstream housing schemes would be highly beneficial in gaining a deeper understanding of differing social inclusion practices across varying socio-sexual configurations.

## Data availability statement

The datasets presented in this study can be found in online repositories. The names of the repository/repositories and accession number(s) can be found below: https://reshare.ukdataservice.ac.uk/855629/.

## Ethics statement

The studies involving human participants were reviewed and approved by Faculty of Social Sciences and Law Research Ethics Committee, University of Bristol (reference 94582). The patients/participants provided their written informed consent to participate in this study.

## Author contributions

PW led on drafting full paper and editing final version. BB contributed to background literature and discussion sections. AV and JP led on interviews with older people and thematic analysis and contributed to drafting of findings. AC and RS contributed to the background and discussion sections and commented on findings section. All authors contributed to the article and approved the submitted version.
